# Rapid Development of Unclassified Myeloid Lineage Acute Leukaemia With Trisomy 6 and *U2AF1* Mutation

**DOI:** 10.1111/jcmm.70461

**Published:** 2025-03-11

**Authors:** Miroslaw Markiewicz, Agnieszka Kopacz, Beata Blajer‐Olszewska, Malwina Mazur, Katarzyna Warzybok, Marta Szarawarska, Marzena Wojtaszewska, Monika Moskwa, Dominika Dudycz, Ewa Schwarz, Katarzyna Kosior, Krzysztof Lewandowski

**Affiliations:** ^1^ Department of Hematology, Institute of Medical Sciences, College of Medical Sciences University of Rzeszow Rzeszow Poland; ^2^ Hematology Department Frederic Chopin University Clinical Hospital Rzeszow Poland; ^3^ Clinic of Tuberculosis and Lung Diseases Frederic Chopin University Clinical Hospital Rzeszow Poland; ^4^ Clinical Department of Pathomorphology Frederic Chopin University Clinical Hospital Rzeszow Poland; ^5^ Faculty of Medicine, Division of Laboratory Medicine Medical University of Gdansk Gdansk Poland

**Keywords:** AML‐molecular diagnosis & therapy, cytogenetics, immunophenotype, myelopoiesis

## Abstract

We present a case of acute clonal bone marrow 98% infiltration of atypical myeloid cells with borderline hypogranular/agranular promyelocytes/myelocytes and occasional blast cells maturity, which also formed extramedullary tumours in the chest wall, with isolated trisomy of chromosome 6 and pathogenic variant *U2AF1* (S34F) that escapes established acute myeloid leukaemia (AML) diagnostic criteria according to the World Health Organization (WHO) classification. Following standard daunorubicin and cytarabine induction therapy, the disease progressed with the appearance of a previously undetected clone of leukaemic cells with a distinct immunophenotype demonstrating monocytoid differentiation and clonal evolution to a hypo‐tetraploid karyotype with an average number of 84 chromosomes and new pathogenic *NRAS* and *ZRSR2* mutations. The patient reactivated refractory disseminated intravascular coagulation (DIC) leading to a progressive supratentorial hematoma and finally cardiac arrest. In conclusion, our report shows that atypical clonal myelocytes can massively infiltrate the bone marrow and form extramedullary tumours, justifying the diagnosis and treatment of acute leukaemia, although they did not fit the current classification.

## 1 Introduction

Successive editions of the WHO classification of hematolymphoid neoplasms, based on a combination of morphological (cytological and histological), immunophenotypic, molecular and cytogenetic data, allow the diagnosis of an increasing number of malignant tumour types and subtypes. However, although improved definition of diagnostic criteria provides increasing potential for classification, it is still possible for a disease to escape the eligibility criteria for a particular category. In such a scenario, diagnosis is extremely difficult and, despite a detailed assessment of the patient, requires extensive diagnostic work‐up.

## 2 Materials and Methods

We present the case of a 61‐year‐old man who developed acute myeloid hyperplasia that escapes established diagnostic criteria. A combination of cytologic and histologic examinations, multiparametric flow cytometry with a broad panel of antibodies and genetic methods were used for the recognition of cells and diagnosis in the context of recently updated diagnostic guidelines. Genetic analyses included the detection of chromosomal abnormalities by conventional cytogenetic analysis and the identification of mutations with the use of polymerase chain reaction (PCR) and next‐generation sequencing (NGS) gene panels to screen for genetic alterations currently mandatory in the evaluation of AML. Detailed analyses were performed at diagnosis and at relapse, allowing for the evaluation of clonal evolution.

## 3 Results

A 61‐year‐old man presented to the Department of Hematology because of unexplained bicytopenia: leukopenia of 2 × 10^9^/L and moderate normocytic anaemia, evolving for about 2 months. The patient had a history of ankylosing spondylitis for 40 years but had received only symptomatic treatment with ad hoc analgesics and rehabilitation and had not received causal treatment for several years. Approximately 2 months prior to hospitalisation, he had been treated with non‐steroidal anti‐inflammatory drugs and two antibiotics for symptoms of respiratory tract infection, mainly dry cough without fever, followed by wandering chest and upper limb pain. Due to his worrying symptoms, the patient underwent laboratory tests ordered by his family doctor, which showed elevated inflammatory markers, increased lactate dehydrogenase (LDH) and bicytopenia. On admission, the patient was in good general condition, complaining only of pain in the lower rib region and had no abnormalities on physical examination. The initial blood count showed moderate anaemia (haemoglobin 92 g/L) and leukopenia (2.21 × 10^9^/L) with neutropenia (0.76 × 10^9^/L), the platelet count was normal. The peripheral blood smear contained 62% lymphocytes, 30% segmented neutrophils, 4% monocytes, and 2% each of myelocytes and metamyelocytes. Neutrophils were mostly normal, with slightly reduced granularity in some.

**FIGURE 1:**
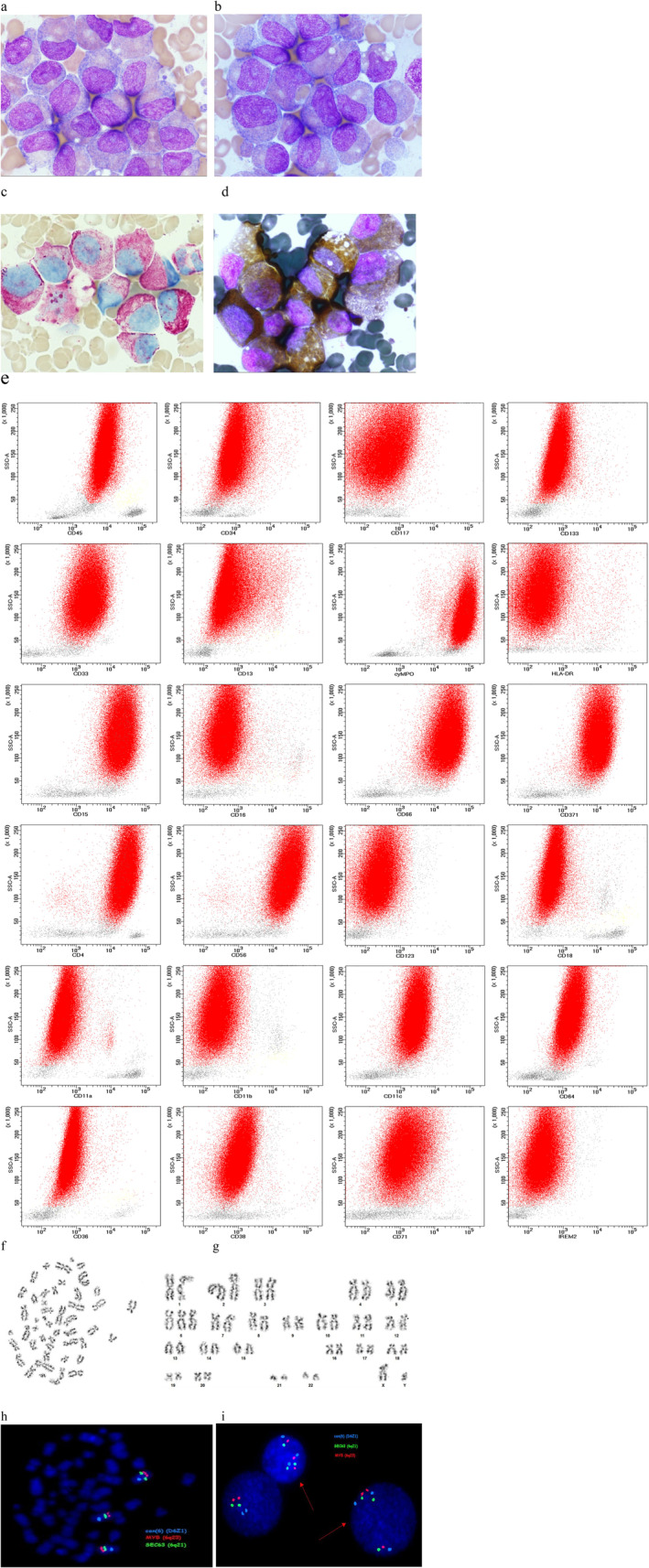
Bone marrow at diagnosis. Panels a, b: Cytology. Hypercellular BM due to massive infiltration of atypical myeloid cells with borderline hypogranular/agranular promyelocytes/myelocytes and occasional blast cells maturity, asynchronism of nuclear‐cytoplasmic maturation with impaired maturation of cytoplasm, reduced granularity and presence of nucleoli. Panels c, d: Cytochemistry. Panel c: Double esterase (DE) staining. Panel d: Myeloperoxidase (MPO) staining. Panel e: Flow cytometry. 98% of abnormal myeloid cells (marked in red). Panels f, g: Karyotype. Isolated trisomy 6 (47,XY,+6). Panels h, i: Fluorescence in situ hybridization with the XL 6q21/6q23/6cen probe (MetaSystems, Altlussheim, Germany) specific for the alfa satellite (centromeric) region (6p11.1‐q11), SEC63 (6q21) and MYB (6q23) showing extra signals in metaphase chromosomes (panel h) and interphase nuclei (panel i), indicating trisomy 6.

Bone marrow aspirates and trephine biopsy histopathology revealed a hypercellular marrow filled with a 98% homogeneous population of atypical myeloid cells with borderline hypogranular/agranular promyelocytes/myelocytes and occasional blast cells maturity (Figure 1, panels a, b). Their cytoplasm showed impaired maturation and reduced granularity (both primary and secondary), while most of the nuclei contained nucleoli. Despite the dysplastic changes observed in the atypical infiltrating cells, there were no clear features of dysplasia in the few remaining cells (e.g., segmented neutrophils and megakaryocytes), which could suggest a dysplastic process originating from low‐differentiated precursors. Megakaryocytes, without significant dysmorphia, were proportional to the cellularity of haematopoiesis, and reticulin fibrosis grade 1, evaluated according to the European Consensus Method [1], was present in the bone marrow stroma. Furthermore, clonal maturity was limited to only two stages (promyelocyte/myelocyte), which was also highly unusual for MDS. The cytopenic nature of the process ruled out myeloproliferative or myeloproliferative/myelodysplastic disease; although, considering morphology, an atypical chronic myeloid leukaemia or another form of mixed syndrome MDS/MPN could be evoked. More mature (metamyelocytes and older) and younger granulocytic forms (myeloblasts and promyelocytes) were almost absent. Erythropoiesis and megakaryopoiesis were completely absent. In cytochemical staining, atypical cells were strongly positive for myeloperoxidase and specific esterase (AS‐D‐chloroacetate) and negative for non‐specific esterase (alpha‐naphthyl acetate) (Figure 1, panels c, d). The specific esterase reaction, in particular, showed Auer rod‐like structures, which were also visible, albeit faintly, in peroxidase smears.

Bone marrow analysis by flow cytometry showed a 98% infiltration of homogeneous, atypical cells with a complex structure (high SSC signal) (Figure 1, panel e). The abnormal population showed expression of myeloid differentiation antigens. Particularly prominent was the absence of immaturity markers (CD34‐, CD117‐, CD133‐, HLA‐DR‐, CD38‐). Features of asynchronous maturation (CD45 + weak, CD33 + weak, CD13‐, CD371 + bright, CD66 + bright, cMPO+bright, CD15 + bright, CD16‐) and abnormal antigen expression (CD56 + high, CD4+) were present. The study population did not show features of monocytic assignment (CD36‐, CD64‐/±, CD11b‐, CD11c‐/±, CD18‐), dendritic cell differentiation was excluded (CD123‐, CD304‐).

Cytogenetic analysis was performed on unstimulated and stimulated bone marrow cultures using standard cytogenetic techniques. Karyotype analysis of 30 metaphases revealed isolated trisomy of chromosome 6 (Figure 1, panels f‐g) in 22 metaphases (73%), while the remaining eight metaphases showed a normal karyotype (ISCN: 47,XY,+6 [22]/46,XY [8]). Metaphase and interphase fluorescence in situ hybridization (FISH) targeting the canonical rearrangements *KMT2A*, *PML*::*RARA*, *CBFB*::*MYH11*, *RUNX1*::*RUNX1T1* showed negative results. The presence of chromosome 6 trisomy was confirmed with CCP6/SEC63/MYB probes by FISH in 1 metaphase (Figure 1, panel h) and in 89/100 interphase nuclei (Figure 1, panel i) [presence of 3 centromere signals of chromosome 6 and *SEC63*(6q21) and *MYB*(6q23) loci]. Molecular evaluation of bone marrow and infiltrated peripheral blood by standard PCR and targeted NGS identified the only pathogenic variant *U2AF1* p.(Ser34Phe) (S34F) with variant allele frequency (VAF) of 46%. In addition, no fusion genes relevant to bone marrow neoplasms were detected in the bone marrow (see Table 1 for full molecular analyses).

**TABLE 1:** Molecular bone marrow testing.

(a) The list of fusion genes tested by PCR at diagnosis			
The tests were performed using Leukaemia (Q30) Fusion Genes Screening Kit (CE‐IVD, ZEESAN). The panel includes the following fusion genes:			
*KMT2A*::*MLL3*	*BCR*::*ABL1*	*SET*::*NUP214*	*NPM1*::*MLF1*
*PML*::*RARA*	*STIL*::*TAL1*	*DEK*::*NUP214*	*NPM1*::*RARA*
*RUNX1*::*RUNX1T1*	*KMT2A*:: *MLLT10*	*KMT2A*::*SEPTIN6*	*KMT2A*::*AF1q*
*KMT2A*::*AFF1*	*B*::*MYH11*	*ETV6*::*PDGRFB*	*RUNX1*::*CBFA2T3*
*RUNX1*::*ETV6*	*RUNX1*::*‐1L1MECOM*	*TLS*::*ERG*	*ETV6*::*ABL1*
*TCF3*::*PBX1*	*FIP1L1*::*PDGRA*	*KMT2A*::*ELL*	*KMT2A*::*AFDN*
*KMT2A*::*MLLT1*	*TCF3*:: *HLF*	*KMT2A*::*MLLT6*	*RUNX1*::*EAP*

(b) The results of NGS at diagnosis			
The targeted amplicon—based NGS panel was performed on genomic DNA isolated from a representative bone marrow sample. The VariantPlex CoreMyeloid (ArcherDx) with molecular barcodes (UMIs) was sequenced on Illumina MiSeqDX with a mean coverage of 2903 X			
Pathogenic or likely pathogenic variant of the following gene was detected:			
*U2AF1* p.(Ser34Phe), VAF: 46%			
No clinically relevant variants of the following genes were detected:			
*ABL1*	*DDX41*	*IDH2*	*RUNX1*
*ANKRD26*	*DNMT3A*	*JAK2*	*SETBP1*
*ASXL1*	*ETNK1*	*KIT*	*SF3B1*
*BCOR*	*ETV6*	*KRAS*	*SRSF2*
*BRAF*	*EZH2*	*MPL*	*STAG2*
*CALR*	*FLT3*	*NPM1*	*TET2*
*CBL*	*GATA1*	*NRAS*	*TP53*
*CEBPA*	*GATA2*	*PFH6*	*WT1*
*CSF3R*	*IDH1*	*PTPN11*	*ZRSR2*

(c) The results of NGS at progression			
The targeted amplicon—based NGS panel was performed on genomic DNA isolated from a representative bone marrow sample. The VariantPlex CoreMyeloid (ArcherDx) with molecular barcodes (UMIs) was sequenced on Illumina MiSeqDX with a mean coverage of 863 X			
Pathogenic or likely pathogenic variants of the following genes were detected:			
*U2AF1* NM_006758.3:c.101C> T p.(Ser34Phe), VAF: 24%			
*NRAS* NM_002524.5: c.181C> A p.(Gln61Lys), VAF: 40%			
*ZRSR2* NM_005089.4:c.1062_1091delinsGTGTCTCCAGATCGGACTGGCTCCTCCTTTCGGAGTTCTTCCCAAAGGA p.(?), VAF: 6% VAF:6%			
No clinically relevant variants of the following genes were detected:			
*ABL1*	*DDX41*	*IDH2*	*RUNX1*
*ANKRD26*	*DNMT3A*	*JAK2*	*SETBP1*
*ASXL1*	*ETNK1*	*KIT*	*SF3B1*
*BCOR*	*ETV6*	*KRAS*	*SRSF2*
*BRAF*	*EZH2*	*MPL*	*STAG2*
*CALR*	*FLT3*	*NPM1*	*TET2*
*CBL*	*GATA1*	*PFH6*	*TP53*
*CEBPA*	*GATA2*	*PTPN11*	*WT1*
*CSF3R*	*IDH1*		

Over a period of about 2 weeks, the blood count gradually decreased, reaching nadir values: haemoglobin 84 g/L, WBC 1.36 × 10^9^/L with absolute neutrophil count (ANC) 0.48 × 10^9^/L and platelet count 62 × 10^9^/L. The patient's clinical condition was relatively good throughout the follow‐up period, except for a single episode of fever of unknown origin, which was quickly controlled with the introduction of anti‐infective drugs. Consequently, due to the lack of an established diagnosis, no targeted treatment was implemented at that time. Over the following 1.5 weeks, blood counts improved spontaneously and the patient was discharged with a haemoglobin level of 100 g/L, a WBC of 2.20 × 10^9^/L with an ANC of 1.12 × 10^9^/L and a normal platelet count. A further outpatient follow‐up 2 weeks later showed further improvement in CBC parameters.

**FIGURE 2:**
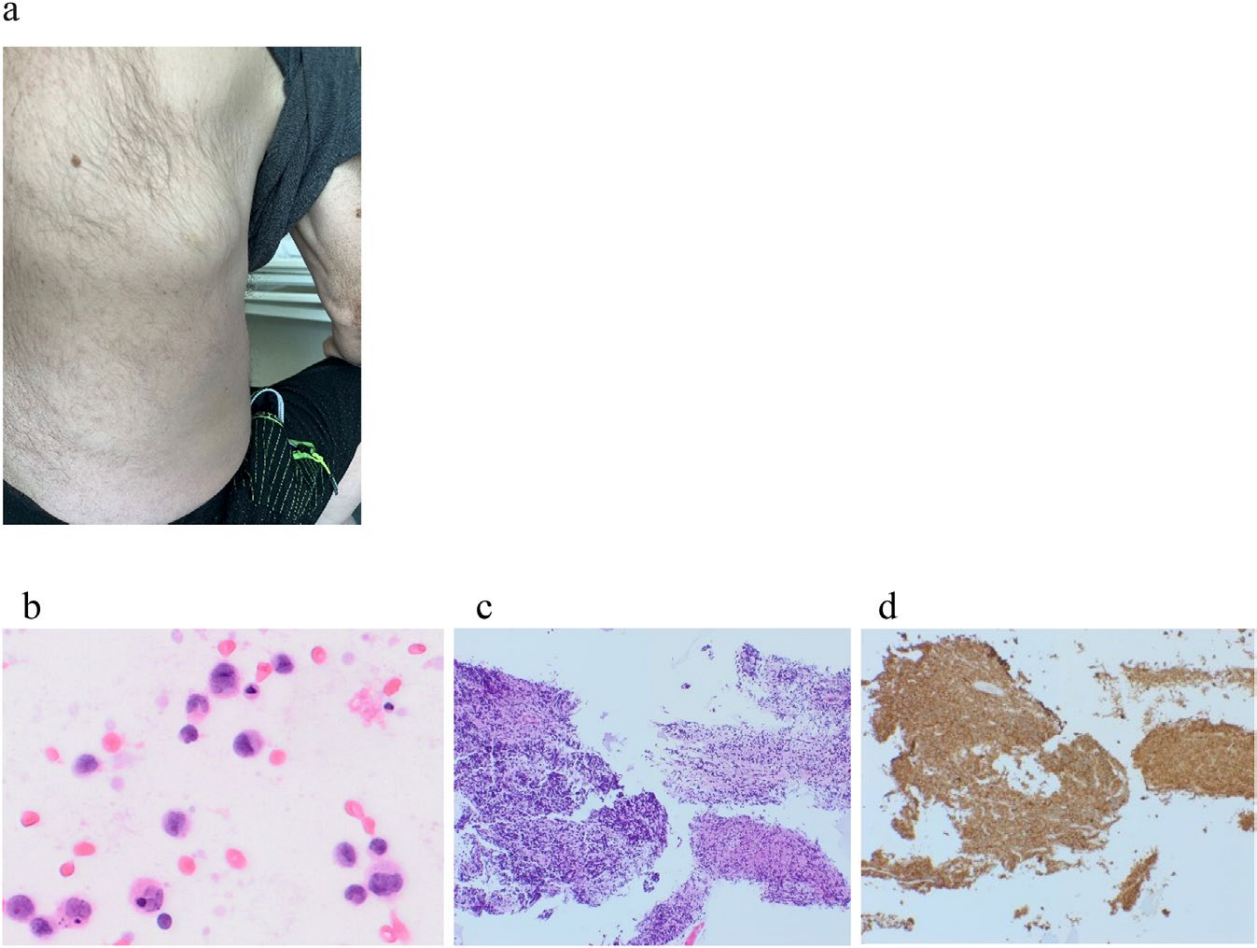
Chest wall tumour at progression. Panel a: Clinically apparent tumour. Panels b–d: Infiltration with atypical myeloid cells in May‐Grunwald Giemsa (MGG, panel b), Haematoxylin and eosin (HE, panel c) and Myeloperoxidase (MPO, panel d) staining.

Ten days later, the patient was unexpectedly admitted to the emergency room for dyspnoea. On auscultation, there was a weakened respiratory murmur on the left side at the base. Chest X‐ray showed uniform shadowing of the middle and lower fields of the left lung, indicating atelectatic changes, and fluid in the left pleural cavity extending to the level of the posterior parts of ribs VII and VIII. There was shadowing on the right side projecting into the chest wall structures. A chest ultrasound confirmed a large amount of fluid on the left side, and on a CT scan of the chest without and with contrast, the layer of fluid in the left pleural cavity was estimated at approximately 8 cm, with accompanying fluid, compressive, increased atelectatic changes in the parenchyma of the left lung, mainly in the lower lobe. A puncture of the left pleural cavity was performed and 1600 mL of serous fluid was evacuated. A further CT scan of the chest showed a pathological mass in the right chest wall, measuring approximately 3.5 cm × 4.5 cm, located posterior‐laterally at the level of ribs VII/VIII, infiltrating the pleura and the oblique fissure, with a local pathological seepage reaction in the structure of the aforementioned ribs. This mass formed a tumour, which later became visible on the chest wall (Figure 2, panel a). In addition, quite numerous smaller thickenings/infiltrates of a similar nature were found scattered in the chest wall along the ribs and in the mediastinum along the spine—the largest in the anterior segment of the right rib of group VI P approximately 1.5 cm. A transthoracic fine‐needle aspiration biopsy (18G) of the chest wall/right lung tumour was performed under CT guidance. Histopathological examination with immunohistochemistry revealed very numerous atypical cells with variable morphology: some with sparse granular cytoplasm, round nuclei with apparent nucleolus, others larger with more acidophilic cytoplasm, eccentric nuclei, and a high degree of atypia (Figure 2, panels b‐d). All (100%) atypical cells were MPO positive, some with slight CD33 positivity. According to the clinical data, the lesion pattern corresponded to a myeloid neoplasm infiltrate, morphologically most similar to promyelocytes/myelocytes (Table 2).

**TABLE 2:** Result of histopathological evaluation with immunohistochemistry of right lung peripleural tumour from CT‐guided biopsy at progression.

Very numerous atypical cells of variable morphology: part with sparse‐granular cytoplasm, round nuclei with nucleolies, other larger with more acidophilic cytoplasm with excentric nuclei and large degree of atypia. All (100%) atypic cells present positive MPO activity, some with slight CD33 positivity. In concordance with clinical data, the picture presents myeloid neoplasm infiltration, morphologically closest to promyelocytes/myelocytes
CD20‐, CD3‐, CD34‐, CD4+, CK PAN—

After extramedullary involvement of the myeloid neoplastic tumour was detected, the patient was readmitted to the haematology department. Blood tests on readmission revealed pancytopenia: haemoglobin 88 g/L, WBC 2.65 × 10^9^/L with ANC 0.5 × 10^9^/L, and platelet count 85 × 10^9^/L. The bone marrow was infiltrated with 31% atypical cells, significantly less compared to 98% at initial assessment; remaining cells were mostly segmented neutrophils, erythroblasts and lymphocytes. The spontaneous reduction in bone marrow infiltration was confirmed by FISH by detecting chromosome 6 trisomy in 41/100 instead of the previously observed 89/100 interphase cells. Flow cytometry was then performed, demonstrating minor changes in immunophenotype. Compared with results observed 3 months earlier, the abnormal cells showed increased expression of CD33, CD13, CD38 and CD64, but decreased expression of CD11c. On whole‐body computed tomography, the size of the pathological hyperplastic chest wall mass located posterior‐laterally at the level of ribs VII/VIII increased (previously 42 × 36 mm, estimated at 61 × 48mm on second exploration) and infiltrated the pleura and oblique fissure over a greater length, and numerous other pathological pleural wall tissue masses also increased, with a localised pathological infiltrative reaction visible within the ribs.

Atypical clonal myeloid cells infiltrating not only the bone marrow, but also forming extramedullary tumours in the chest wall, undoubtedly presented malignant findings mandating a diagnosis of myeloid neoplasm similar to acute myeloid leukaemia, with a *U2AF1* mutation defining AML‐associated myelodysplasia, although the patient did not fit the WHO 2022 classification as AML, myelodysplasia‐related (AML‐MR) due to a more advanced maturation stage than myeloblast/promelocyte and no history of MDS or MDS/MPN [2]. Therefore, the patient was considered to have unclassified acute myeloid leukaemia and standard induction therapy with daunorubicin and cytosine arabinoside (DA) was administered. During treatment, transient bleeding and laboratory features of DIC were observed; with the exception of this and a single episode of fever, tolerability was good. After a period of post‐treatment aplasia, platelet recovery > 50 × 10^9^/L occurred on day +21 and neutrophils > 1 × 10^9^/L on day +27, and the patient was discharged home.

**FIGURE 3:**
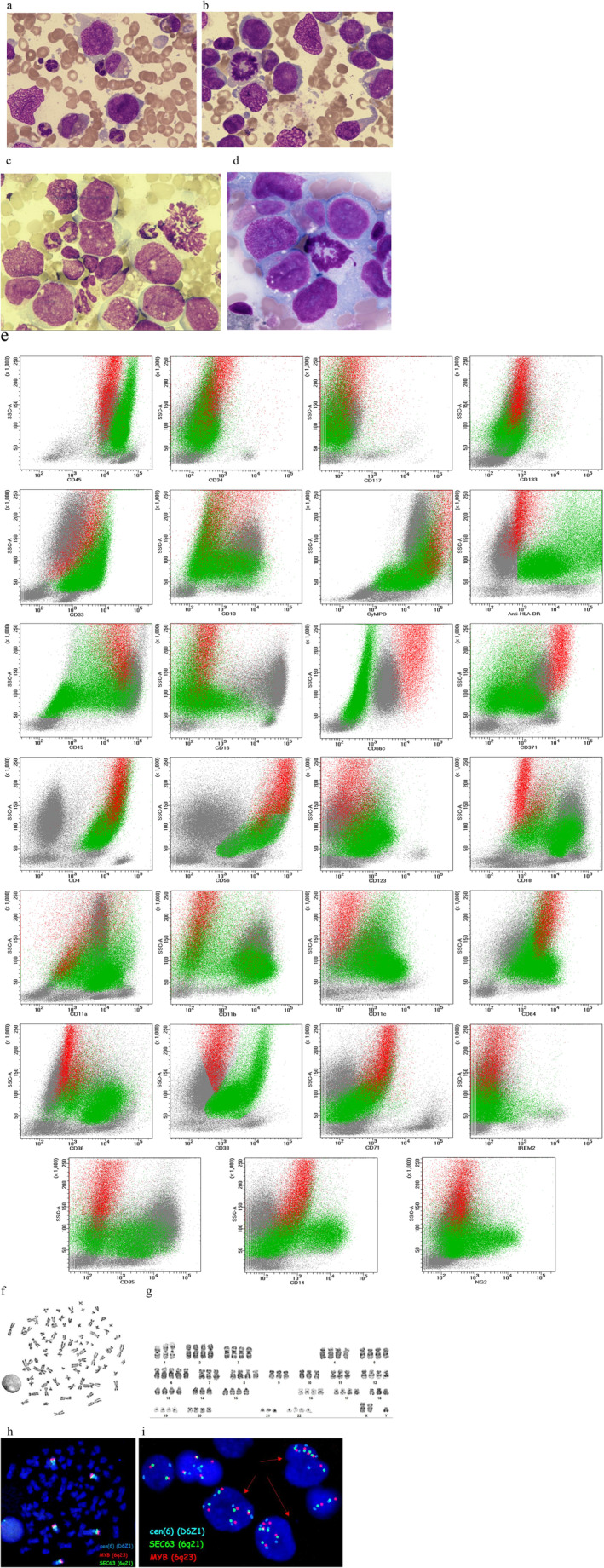
Bone marrow at progression. Panels a, b, c and d: Cytology. Infiltration of atypical megaloblastic cells. Panel e: Flow cytometry. Two abnormal populations: Myeloid, previously observed (marked in red) and monocytoid, newly detected (marked in green). Panels f‐g: Karyotype. Metaphase prepared for FISH (panel f), hypo‐tetraploid karyotype with 84 chromosomes (panel g). Panels h‐i: Detection of chromosomal changes using FISH showing multiple extra signals in metaphase chromosomes (panel h) and interphase nuclei (panel i).

After a further 5 days, the patient was readmitted due to severe weakness, dyspnoea and the appearance of bloody fluid in the left pleural cavity drain. On admission, the patient was in poor general condition, ECOG 4, lethargic, confused and intermittently illogical. Morphology showed hyperleukocytosis reaching 74.81 × 10^9^/L with a leftward shift towards myeloblasts (30%). Bone marrow cytology revealed a hypercellular marrow with 49.0% atypical immature megaloblastic cells, some flare around the nucleus, fine cytoplasmic granulations, polymorphous/multiform nuclei and disrupted/disconnected cytoplasm (Figure 3, panels a–d). Bone marrow flow cytometry confirmed the progression of the disease and revealed the presence of two abnormal leukaemic cell populations: one similar to that previously described, representing approximately 8% of all nucleated cells, and the appearance of a larger, previously undetected clone of leukaemic cells with a distinct immunophenotype (Figure 3, panel e). No immature markers were detected and the newly noticed population expressed markers of monocytoid lineage (CD33 + bright, CD13+/−, HLA‐DR+, MPO+, CD15+/−, CD64+, CD36+, CD14+/−, CD4+, CD18 + bright, CD11a + bright, CD11b+/−, CD11c+, CD38+). In addition, this population was positive for CD56 (obviously on a subset) and CD123. Cytogenetic evaluation showed clonal evolution to a hypo‐tetraploid karyotype with an average number of 84 chromosomes (84,XXY,‐Y,‐1,+6,‐7,‐9,‐10,‐11,‐14,‐18,‐21[21]/46,XY [9]) (Figure 3, panels f, g). The presence of complex chromosomal abnormalities was confirmed by FISH in metaphase and interphase cells (Figure 3, panels h, i). Pathogenic variants specific for MDS‐type myeloid neoplasms were found by NGS in the *NRAS*, *U2AF1* and *ZRSR2* genes with VAFs of 40%, 24% and 6%, respectively. Compared to the sample at diagnosis, new pathogenic *NRAS* and *ZRSR2* mutations were selected.

A follow‐up CT scan of the head was performed to complete the diagnosis of impaired consciousness, revealing a progressive supratentorial hematoma, but due to his general condition and coexisting coagulation disorders associated with reactivated refractory DIC, the patient was not qualified for neurosurgical intervention. Despite intensive supportive treatment, there was no improvement in the general condition or return of consciousness, and the patient was pronounced dead due to cardiac arrest.

## 4 Discussion

The case we present illustrates the puzzling clinical features associated with pathological cells with a degree of differentiation beyond the promyelocyte stage, with chromosome 6 trisomy and *U2AF1* mutation, forming a massive bone marrow infiltrate and extramedullary mass in the chest wall and lungs. To our knowledge, no similar case has been described to date.

In the recently published results of a meta‐analysis, the authors identified a large cohort of 78 patients with isolated trisomy 6 and bone marrow tumours (48 with AML, 16 with MDS) and 14 with aplastic anaemia (AA). The results suggest that the role of trisomy 6, the chromosome containing the major tissue compatibility complex encoding the HLA system, may be context‐dependent. In AA and hypocellular MDS, increased expression of HLA peptides may lead to increased TCR activation, triggering an immune response towards haematopoietic cells which was manifested by pancytopenia and bone marrow hypocellularity. In AML, the risk may be unfavourable, related to immune pressure with somatic loss of HLA: lack of increased HLA expression due to HLA downregulation, allelic deletion of HLA genes or microdeletion of tumour suppressor genes may contribute to immune escape, hyperproliferation and more advanced leukaemia [3].

U2 small nuclear RNA accessory factor 1 (*U2AF1*) is a multifunctional protein that plays a key role in regulating RNA splicing after gene transcription. Mutations in the *U2AF1* gene have been described in a wide range of cancers, including myelodysplastic syndrome, acute myeloid leukaemia, primary myelofibrosis, chronic myelomonocytic leukaemia, hairy cell leukaemia and various solid tumours [4]. S34F, found in the present case, is one of the major *U2AF1* mutation sites and the most frequently observed (60%) [5]. Mutations of spliceosome genes, including *U2AF1*, have the highest predictive value for bone marrow malignancies, regardless of co‐occurring mutations, in patients with unexplained cytopenias [6].

Currently, the prognosis of *U2AF1* mutations in cancer, particularly in haematological diseases, remains to be fully elucidated. Cells expressing the *U2AF1* S34F gene had defective autophagy along with mitochondrial dysfunction and increased production of reactive oxygen species, causing genome instability and an increased frequency of spontaneous mutations, which can interfere with metabolism, inflammation, genome stability and control of cell proliferation [7]. Mutant *U2AF1* has been shown in an animal model to alter the expression of downstream gene isoforms, thereby contributing to abnormal haematopoiesis [8].

At the time of progression, a loss‐of‐function variant in the *ZRSR2* gene was detected. The *ZRSR2* protein is another factor affecting splicing regulation that is particularly observed in AML arising after MDS or MDS/MPN [2]. Unlike *U2AF1*, one of the three most commonly mutated components of the major spliceosome, *ZRSR2* is a component of the minor spliceosome and often exhibits a loss‐of‐function effect [9]. *U2AF1* and *ZRSF2* rarely co‐occur and are considered mutually exclusive in bone marrow tumours [10], suggesting that either both variants may have occurred in different clones during the clonal evolution of the disease, or spliceosomal regulation in cells carrying both mutations has been severely altered. A second novel mutation, the *NRAS* canonical hotspot variant at codon 61, was correlated with more aggressive disease and reduced survival in MDS [11].

Very rare cases of a rearrangement in the *RARG* or *RARB* genes have been reported in patients with acute promyelocytic leukaemia [12]. Unfortunately, there are no commercially available FISH probes for these genes and thus we were not able to exclude them by FISH testing; however, their involvement in the present case seems extremely unlikely.

## 5 Conclusion

Our report shows that atypical myelocytes can massively infiltrate the bone marrow and form extramedullary tumours, which, together with the genetic confirmation of their clonality through the detection of *U2AF1* mutations and chromosome 6 trisomy, justified the diagnosis and treatment of acute leukaemia, which did not fit the current classification. However, the interplay between the abnormal splicing machinery and the extra chromosome 6 has not yet been established as sufficient for the development of the presented phenotype and the aggressive course of the disease.

## Author Contributions

**Miroslaw Markiewicz:** conceptualization (lead), data curation (lead), formal analysis (lead), investigation (lead), methodology (lead), project administration (lead), supervision (lead), visualization (lead), writing – original draft (lead), writing – review and editing (lead). **Agnieszka Kopacz:** investigation (equal), methodology (equal), resources (equal). **Beata Blajer‐Olszewska:** investigation (equal), methodology (equal), resources (equal). **Malwina Mazur:** investigation (equal), resources (equal). **Katarzyna Warzybok:** data curation (equal), resources (equal). **Marta Szarawarska:** methodology (equal), resources (equal), validation (equal). **Marzena Wojtaszewska:** methodology (equal), resources (equal), validation (equal), writing – review and editing (equal). **Monika Moskwa:** methodology (equal), validation (equal). **Dominika Dudycz:** methodology (equal), resources (equal). **Ewa Schwarz:** investigation (equal), resources (equal). **Katarzyna Kosior:** resources (equal). **Krzysztof Lewandowski:** investigation (equal), methodology (equal), supervision (equal), validation (equal), writing – review and editing (equal).

## Conflicts of Interest

The authors declare no conflicts of interest.

## Data Availability

Data sharing is not applicable to this article as no new data were created or analyzed in this study.

## References

[1] J. Thiele , H. M. Kvasnicka , and F. Facchetti , “European Consensus on Grading Bone Marrow Fibrosis and Assessment of Cellularity,” Haematologica 90 (2005): 1128–1132. 16079113

[2] J. D. Khoury , E. Solary , O. Abla , et al., “The 5th Edition of the World Health Organization Classification of Haematolymphoid Tumors: Myeloid and Histiocytic/Dendritic Neoplasms,” Leukemia 36 (2022): 1703–1719. 35732831 10.1038/s41375-022-01613-1 PMC9252913

[3] H. Awada , A. Durmaz , T. Kewan , et al., “Context‐Dependent Role of Trisomy 6 in Myelodysplastic Neoplasms and Acute Myeloid Leukemia: A Multi‐Omics Analysis,” Leukemia 38 (2024): 1411–1414. 38734787 10.1038/s41375-024-02268-w PMC11147751

[4] Q. Nian , Y. Li , J. Li , et al., “U2AF1 in Various Neoplastic Diseases and Relevant Targeted Therapies for Malignant Cancers With Complex Mutations (Review),” Oncology Reports 51, no. 1 (2024): 5. 37975232 10.3892/or.2023.8664 PMC10688450

[5] T. Badar , Y. A. M. Vanegas , A. Nanaa , et al., “U2AF1 Pathogenic Variants in Myeloid Neoplasms and Precursor States: Distribution of Co‐Mutations and Prognostic Heterogeneity,” Blood Cancer Journal 13, no. 1 (2023): 149. 37735430 10.1038/s41408-023-00922-7 PMC10514309

[6] L. Malcovati , A. Gallì , E. Travaglino , et al., “Clinical Significance of Somatic Mutation in Unexplained Blood Cytopenia,” Blood 129, no. 25 (2017): 3371–3378, 10.1182/blood-2017-01-763425. 28424163 PMC5542849

[7] S. M. Park , J. Ou , L. Chamberlain , et al., “U2AF35(S34F) Promotes Transformation by Directing Aberrant ATG7 Pre‐mRNA 3′ End Formation,” Molecular Cell 62 (2016): 479–490. 27184077 10.1016/j.molcel.2016.04.011 PMC5012111

[8] C. L. Shirai , J. N. Ley , B. S. White , et al., “Mutant U2AF1 Expression Alters Hematopoiesis and Pre‐mRNA Splicing in Vivo,” Cancer Cell 27, no. 5 (2015): 631–643. 25965570 10.1016/j.ccell.2015.04.008 PMC4430854

[9] I. M. Bouligny , K. R. Maher , and S. Grant , “Secondary‐Type Mutations in Acute Myeloid Leukemia: Updates From ELN 2022,” Cancers 15, no. 13 (2023): 3292. 37444402 10.3390/cancers15133292 PMC10340017

[10] Y. S. Kwon , S. W. Jin , and H. Song , “Global Analysis of Binding Sites of U2AF1 and ZRSR2 Reveals RNA Elements Required for Mutually Exclusive Splicing by the U2‐ and U12‐Type Spliceosome,” Nucleic Acids Research 52, no. 3 (2024): 1420–1434. 38088204 10.1093/nar/gkad1180 PMC10853781

[11] D. Alawieh , L. Cysique‐Foinlan , C. Willekens , and A. Renneville , “RAS Mutations in Myeloid Malignancies: Revisiting Old Questions With Novel Insights and Therapeutic Perspectives,” Blood Cancer Journal 14 (2024): 72. 38658558 10.1038/s41408-024-01054-2 PMC11043080

[12] M. C. Geoffroy , C. Esnault , and H. de Thé , “Retinoids in Hematology: A Timely Revival?,” Blood 137, no. 18 (2021): 2429–2437. 33651885 10.1182/blood.2020010100

